# Modeling Full-Field Transient Flexural Waves on Damaged Plates with Arbitrary Excitations Using Temporal Vibration Characteristics

**DOI:** 10.3390/s22165958

**Published:** 2022-08-09

**Authors:** Dan-Feng Wang, Kuo-Chih Chuang, Jun-Jie Liu, Chan-Yi Liao

**Affiliations:** 1 Key Laboratory of Soft Machines and Smart Devices of Zhejiang Province, School of Aeronautics and Astronautics, Institute of Applied Mechanics, Zhejiang University, Hangzhou 310027, China; 2 Applied Mechanics and Structure Safety Key Laboratory of Sichuan Province, School of Mechanics and Aerospace Engineering, Southwest Jiaotong University, Chengdu 610031, China; 3 Department of Mechanical Engineering, National Central University, Taoyuan 32001, China

**Keywords:** flexural waves, vibrations, structural health monitoring, semi-analytical method

## Abstract

We propose an efficient semi-analytical method capable of modeling the propagation of flexural waves on cracked plate structures with any forms of excitations, based on the same group of vibration characteristics and validated by a non-contact scanning Laser Doppler Vibrometer (LDV) system. The proposed modeling method is based on the superposition of the vibrational normal modes of the detected structure, which can be applied to analyze long-time and full-field transient wave propagations. By connecting the vibration-based transient model to a power flow analysis technique, we further analyze the transient waves on a cracked plate subjected to different excitation sources and show the influence of the damage event on the path of the propagating waves. The experimental results indicate that the proposed semi-analytical method can model the flexural waves, and through that, the crack information can be revealed.

## 1. Introduction

Structural health monitoring (SHM) technologies are crucial in maintaining the safety and integrity of engineering structures and thus have attracted much attention in the research community [1,2,3]. Generally, non-destructive damage detection techniques can be characterized by the vibration-based [4,5] and guided wave-based methods [6,7]. The guided wave-based detection techniques are suitable for sensitively locating small or local damages. However, detected signals in guided waves are liable to be contaminated by environmental noises, such as temperature effects [8,9], dispersion properties [10], multiple modes [11], and material uncertainties [12]. The vibration-based detection techniques, on the other hand, can detect global damage events and have the advantage of requiring no a priori information of the vicinity of damage. The existence or onset of damage will alter the vibration characteristics, such as shifts of natural frequencies, changes of modal damping, or derivatives of mode shapes, of the detected structures, and thus, the vibration characteristics contain the information of the damage events [13,14,15,16]. Generally, the vibration-based and guided wave-based methods are regarded as two distinct approaches despite the fact that vibration and waves are essentially closely related.

Waves propagating in plate structures, known as Lamb waves, are sensitive to various types of damage and their propagation characteristics and interpretation are crucial in developing related monitoring strategies [17,18,19,20]. Numerically, Lamb waves are generally analyzed by the finite element method (FEM) [21,22], finite difference method (FDM) [23,24], the spectral element method (SEM) [25], the boundary element method (BEM) [26], or the semi-analytical finite element method (SAFE) [27,28], where the SAFE has recently been developed to model guided wave on anisotropic composite laminates and in situations with thermal effect [29,30]. Although vibrations- and waves-based damage detection techniques are characterized as two distinct approaches, the vibration can be regarded as standing waves while waves can be regarded as the superposition of vibrational modes. Thus, the damage-induced changes of the modal parameters in the time-domain should contribute to the behavior of the spatial-domain propagating waves. In addition to capturing the wave propagation characteristics with plate structures with cracks, analysis of spatial-domain propagating Lamb waves based on time-domain vibration characteristics enables us to calculate the instantaneous wave responses at any given time under different arrangements or forms of excitations with the same database of vibration information and time-consuming iterative time-stepping process from the initial condition required in general numerical methods can be avoided.

Aiming to provide a modeling method for cracked plate structures by linking the vibration and wave, in this work, we propose a semi-analytical method for modeling the full-field transient propagation of flexural waves (i.e., the approximation of the low-frequency A0 Lamb wave mode) on a cracked plate based on temporal vibration characteristics and the superposition of the deviated vibrational normal modes. The proposed semi-analytical method is further connected to a power flow analysis technique to analyze the transient waves excited by different excitation sources. Finally, the proposed semi-analytical method is validated by a non-contact scanning Laser Doppler Vibrometer (LDV) system.

## 2. Semi-Analytical Model Linking the Vibrations and Waves

Classical plate theory claims that if the coordinate in the thickness direction of a three-dimensional solid can be eliminated, the three-dimensional plate problem can be reduced to a two-dimensional problem and physical quantities, such as displacements, strains, and stresses, at all points in the mid-plane of the plate can be used to determine the associated quantities at any point in the three-dimensional plate structure [31]. When the thin plate has cracks symmetrical in the direction of thickness, where the mid-plane of the plate remains continuous, the classical plate theory can still be applied. Based on the classical plate theory, we now propose a semi-analytical method to analyze the transient behaviors of a cracked plate.

Consider a homogeneous thin plate, also known as the Kirchhoff plate, with an external loading $p\left(x_{1},x_{2},t\right)$ applied on the upper surface of the plate. The governing equation for the out-of-plane deformation $u_{3}$ is [32]

(1) $$D\left(\frac{\partial^{4}u_{3}}{\partial {x_{1}}^{4}}+2\frac{\partial^{4}u_{3}}{\partial {x_{1}}^{2}\partial {x_{2}}^{2}}+\frac{\partial^{4}u_{3}}{\partial {x_{2}}^{4}}\right)+\rho_{s}h\frac{\partial^{2}u_{3}}{\partial t^{2}}=p\left(x_{1},x_{2},t\right),$$

where $D=\frac{Eh^{3}}{12(1-\nu^{2})}$, E is Young’s modulus, $\rho_{s}$ is the density, ν is Poisson’s ratio, and $h\left(x_{1},x_{2}\right)$ is the thickness at position $\left(x_{1},x_{2}\right)$ of a plate. The out-of-plane transient displacement of the plate can be written as a superposition of the vibration normal modes as follows

(2) $$u_{3}\left(x_{1},x_{2},t\right)=\sum_{i=1}^{\infty }T_{i}\left(t\right)W_{i}\left(x_{1},x_{2}\right),$$

where $W_{i}\left(x_{1},x_{2}\right)$ is the *i*th mode shape, $T_{i}\left(t\right)$ is the time function representing the weight coefficients of the contribution of the *i*th mode shape at a specific time to the transient wave field. By the orthogonal properties between the mode shapes in the modal space, the transient displacement solution of the flexural wave (i.e., the approximation of the low-frequency A0 Lamb wave mode) can be derived from Equation (1) as [32]

(3) $$u_{3}\left(x,y,t\right)=\sum_{i=1}^{\infty }\frac{W_{i}\left(x_{1},x_{2}\right)}{\rho_{s}\omega_{i}}\left\{\frac{\int_{0}^{t}\left[\int_{0}^{b}\int_{0}^{a}p\left(x_{1},x_{2},\tau \right)W_{i}dx_{1}dx_{2}\right]\sin\omega_{i}\left(t-\tau \right)d\tau }{\int_{0}^{b}\int_{0}^{a}\left(h\cdot {W_{i}}^{2}\right)dx_{1}dx_{2}}\right\},$$

where $\omega_{i}$ is the resonant frequency of the *i*th resonant mode of the plate and a and b are the length and width of the plate, respectively. If the external loading with a loading history F(t) is applied within a specific range $R\left(x_{1},x_{2}\right)$, Equation (3) can be written as the following Duhamel’s integral

(4) $$u_{3}\left(x,y,t\right)=\sum_{i=1}^{\infty }\left[\frac{W_{i}\left(x_{1},x_{2}\right)\cdot \int_{0}^{b}\int_{0}^{a}R\left(x_{1},x_{2}\right)\cdot W_{i}dx_{1}dx_{2}}{\rho_{s}\omega_{i}\cdot \int_{0}^{b}\int_{0}^{a}\left(h\cdot {W_{i}}^{2}\right)dx_{1}dx_{2}}\right]\cdot \int_{0}^{t}F\left(\tau \right)\sin\omega_{i}\left(t-\tau \right)d\tau .$$

From the term $\int_{0}^{b}\int_{0}^{a}R\left(x_{1},x_{2}\right)\cdot W_{i}dx_{1}dx_{2}$ shown in the temporal solution in Equation (4), we can see that the applying range of the external loading will influence the contribution of the individual mode. Note that $R\left(x_{1},x_{2}\right)$ is expressed as a Dirac-delta function $\delta \left(x_{1}-x_{1p}\right)\delta \left(x_{2}-x_{2p}\right)$ when a pointwise excitation is applied at position $\left(x_{1p},x_{2p}\right)$. Although the out-of-plane displacement is dependent on space and time, the vibration characteristics $\omega_{i}$ and $W_{i}\left(x_{1},x_{2}\right)$ in Equation (4) (i.e., resonant frequencies and mode shapes) are determined by the boundary conditions, material constants, and geometry of the plate and are independent of time, magnitude of the external loadings, and the excitation locations. Thus, even for complicated transient responses, if the vibration characteristics can be obtained beforehand through theoretical calculations or finite element simulations, transient behaviors at any time and any location can be obtained by Equation (4) by the fact that the vibration characteristics and wave source are decoupled and thus can be analyzed separately. We note that the frequencies and mode shapes of the analyzed plate can also be experimentally measured using techniques, such as experimental modal analysis (EMA) or electronic speckle pattern interferometry (ESPI) [33]. However, it should be noted that limited by the bandwidth of the excitations as well as the resolution of the sensing systems, high-frequency resonant frequencies and mode shapes are usually difficult to be experimentally identified. In this work, resonant frequencies and mode shapes of the intact plate are theoretically derived [32]. However, for the damaged plate, vibration characteristics are obtained using finite element analysis.

The analysis of the instantaneous wave propagations on the damaged plate is based on the temporal contributions of each vibrational mode to the overall transient wave field. Although each vibrational mode contributes to the overall transient waves, only a finite number of vibrational modes are required to efficiently converge the calculation. However, when the excitation frequency is high with a short wavelength, more modes are required, and the convergence speed of the calculation would be lower. In addition, for the calculation of the overall transient responses, only a specific range of dominated resonant frequencies centered around the excitation frequency are required. Furthermore, according to the Nyquist sampling theory, when the excitation frequency is at *N* Hz, the considered highest mode in the modal contribution should have resonant frequency higher than 2*N* Hz for the convergence of the transient solution [34].

This semi-analytical approach based on the vibration characteristics enjoys the following advantages:

(1) When the loading range $R\left(x_{1},x_{2}\right)$ or the loading history F(t) is changed, the same vibration characteristics in the database of the vibration information can be substituted by Equation (4) to obtain the transient solutions.
(2) There is no time-consuming iterative time-stepping process in calculating the temporal integral term $\int_{0}^{t}F\left(\tau \right)\sin\omega_{i}\left(t-\tau \right)d\tau$. Thus, we can efficiently obtain on-demand full-field wave propagations at a specific time t.
(3) The form of the transient solution in Equation (4) can be further transformed to other physical quantities, such as strain or velocity fields, without having to perform any additional numerical post-processing operations, which minimizes the possible sacrifice of the resolution (spatial or temporal) or distortion of the transient solutions coming from numerical processing. Based on this advantage, in addition to the relation between the vibration modes and the instantaneous wave responses, in this work, a power flow analysis (PFA) is also considered to reveal the transmission paths of the propagating waves on the plate. The PFA uses the instantaneous power flow density vector defined as the dot product of the velocity vector and stress tensor in the form of $q_{j}=-\sigma_{ji}v_{i}\;,\;i,\;j=1,2,3$, to trace the instantaneous energy transmission paths from one location to another within the plate [35,36,37].

## 3. Simulation of the Instantaneous Flexural Waves Using the Semi-Analytical Model

**Figure 1:**
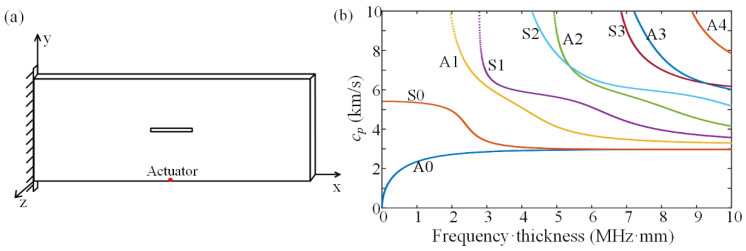
(**a**) Dimension of the damaged plate and the location of the actuator. (**b**) The dispersion curves.

To demonstrate the proposed semi-analytical model for analyzing instantaneous wave propagation, here, we investigate a damaged cantilever rectangular aluminum plate (400×150×2 mm) with a rectangular slot (60×5×2 mm) in the center, which has been studied in the literature, as shown in Figure 1a [4,38]. The isotropic plate is made of aluminum material with a Young’s modulus of 69 GPa, a Poisson’s ratio of 0.33, and a density of 2705 kg/m^3^. The dispersion curves, obtained by the FEM analysis using the COMSOL software for the analyzed plate, are shown in Figure 1b. For the Lamb waves on plates, each frequency corresponds to both the symmetric and asymmetric modes on the dispersion curves. However, by using a windowed excitation signal around a low-dispersion frequency with an asymmetric arrangement of the actuator, a dominated mode (here, the low-frequency A0 mode) can be generated. The transient behaviors of the excited flexural wave (i.e., the low-frequency approximation of the A0 mode) can be analytically captured based on the Kirchhoff plate theory given in Section 2. Later, to model and study the influence of the crack, we considered low-frequency one-sided excitations, and thus, practically, the flexural waves with a high signal-to-noise ratio are mainly excited [39].

Before demonstrating the disturbed waves on the damaged plate, we first consider an intact plate with a point source in the form of a five-cycle tone burst at an operation frequency $f_{0}$ located at coordinate (200 mm, 0 mm, 2 mm) as marked in Figure 1a. The expression of the five-cycle tone burst is as follows:

(5) $$A_{1}(t)=\frac{1}{2}[H(t)-H(t-5/f_{0})]\left[1-\cos(2\pi f_{0}t/5)\right]\sin(2\pi f_{0}t),$$

where H(t) is the Heaviside step function. To avoid numerical errors, Equation (5) will be substituted by the temporal solution in Equation (4) and a closed form of the integral can be obtained.

Then, we show the number of modes required to determine the transient displacement field. We compute the root-mean-square (RMS) of the displacement field ${u_{3}}^{rms}$ at the coordinate (200 mm, 150 mm, 2 mm), with a 1 ms duration and a 1 MHz sampling rate, where

(6) $${u_{3}}^{rms}=\sqrt{\frac{\sum_{i=1}^{M}\left[{u_{3}}^{2}\left(t_{1}\right)+{u_{3}}^{2}\left(t_{2}\right)+{u_{3}}^{2}\left(t_{3}\right)+\ldots {u_{3}}^{2}\left(t_{M}\right)\right]}{M}}.$$

**Figure 2:**
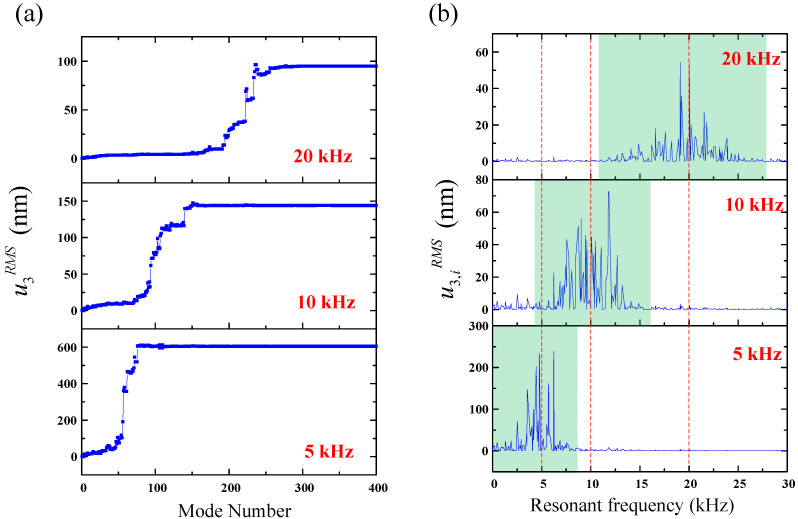
(**a**) The relation between the root-mean-square (RMS) transient displacement and the required number of vibrational modes. (**b**) The relation between the RMS transient displacement due to the *i*th mode and the resonant frequency.

The number of the composed vibration modes with respect to the RMS displacement field at excitation frequencies of 5, 10, and 20 kHz is considered, as shown in Figure 2a. Although only a finite number of vibrational modes is required for converging the solution, the convergent speed of the RMS displacement field is related to the excitation frequency. When the excitation frequency is high, more modes are required for the convergence of the RMS displacement field. Then, we show the contribution of each mode (i.e., ${u_{3,i}}^{rms}$) to the overall transient responses with respect to the resonant frequency in Figure 2b. We can see that as the excitation frequency gets higher, the bandwidth of the contributing modes gets wider, where the dominating modes of the transient responses are centered around the excitation frequency.

**Figure 3:**
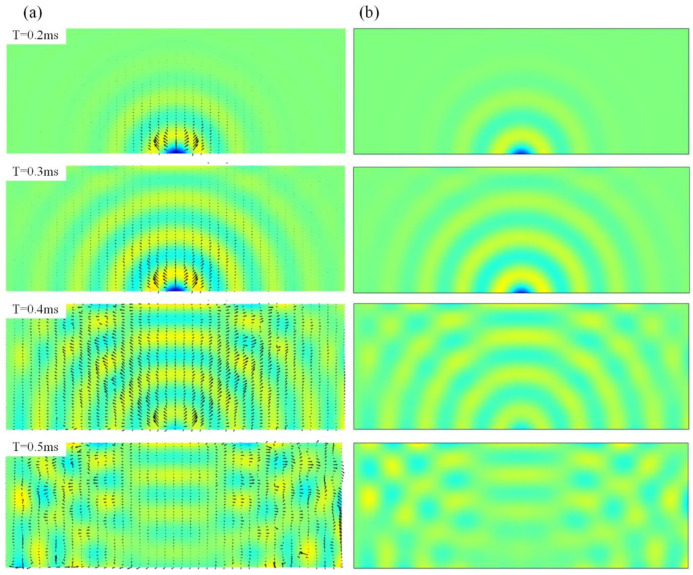
The full-field displacement response within 0.2 to 0.5 ms of the intact plate (**a**) the semi-analytical method with the power transmission paths. (**b**) The FEM methods.

The normalized full-field transient wave fields of the intact plate when the operation frequency is 10 kHz are plotted in Figure 3, where the Solid Mechanics Module with the time-dependent solver is utilized. Good correspondences can be obtained between the semi-analytical method and the FEM results, where the maximum displacements are $4.51\times 10^{-7}\;m$ and $4.57\times 10^{-7}\;m$, respectively. Here, in calculating the semi-analytical method, there are a total of 400 vibrational modes considered, with a span from 0 to 35 kHz. The PFA results that give the energy transport at every location and at every specific time are also listed in Figure 3a, where the arrows demonstrate the instantaneous transmission paths of the propagating waves, and their lengths refer to the intensity of the vectors. We can see that, as time increases, the waves expand from the point source and are finally reflected from the boundary to interfere with the coming waves. Since the boundary condition is clamped at the left end, the wave pattern is not symmetric.

Benefiting from avoiding the time-consuming iterative time-stepping process, the proposed semi-analytical method needs less calculation time compared to the FEM. To compare the simulation efficiency, a computer with the configuration of an AMD Ryzen 7 5800X 8-Core Processor 3.8 GHz and 32 GB RAM was used to run the simulations using the semi-analytical method (i.e., a combination of the FEM for Eigen Frequency Module and the theoretical Duhamel’s integral solution) and the FEM (i.e., time-dependent solver). The mesh of the model consisted of 27,229 elements and the duration of the observation time is 1 ms. The comparisons of the calculation time between the FEM and the proposed semi-analytical method with respect to three different sampling times are shown in Table 1. As the sampling time is decreased, the calculation time for the FEM is increased. However, mainly spent on the obtainment of the vibration modes, the additional calculation time for the semi-analytical method is all very short. For the case of a 1 μs sampling time, the calculation time for the semi-analytical method is only 53% of that used in the FEM analysis.

**Figure 4:**
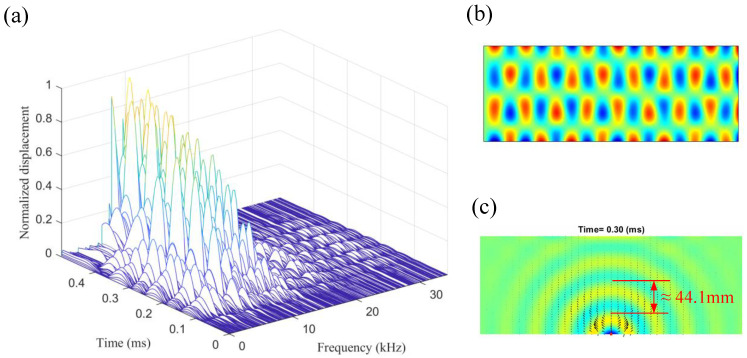
(**a**) The relationship of the displacement of the flexural waves with respect to the time and each vibration mode at its resonant frequency. (**b**) The 129th mode shape around 10 kHz with a resonant frequency of 10,077 Hz (**c**). The full-field displacement response at 0.3 ms, where the flexural wavelength is 44.1 mm.

The relationship of the amplitude of the Lamb waves with respect to the time and each vibration mode contribution at its resonant frequency is given as a three-dimensional plot shown in Figure 4. We can see that, as time passes by, more and more modes are involved in the contribution of the instantaneous wave field. However, although each vibration mode contributes to the overall wave field, only the vibration modes centered around the excitation frequency will dominate the transient wave field, where the amplitude of a specific vibration mode is the largest. Similar to the Nyquist sampling theory, well known in signal analysis, for the convergence of the transient solution, vibrational modes higher than 2*N* Hz are required when the excitation frequency is at *N* Hz. However, from Figure 4, we can see that the dominated vibration modes are within 2*N* Hz [34]. We also note that, even for a specific mode, its contribution to the full-field wave field is not uniform and instead will oscillate as time increases, and the oscillation frequency is 2*N* Hz when the excitation frequency is *N* Hz.

It is interesting to point out that the wavelength of the dominated mode is related to the excitation frequency of the transient wave as

(7) $$\lambda_{f}=\frac{c_{f}}{f}=\sqrt{\frac{2\pi }{f}}{\left[\frac{Eh^{2}}{12\rho_{s}\left(1-\nu^{2}\right)}\right]}^{1/4},$$

where $c_{f}$ is the phase velocity of the flexural wave, which has been given in Figure 1b. By substituting the 10 kHz excitation frequency by Equation (8), we can get a wavelength of 44.1 mm, which is close to the distance between the nodal lines of the dominating mode, the 129th mode, plotted in Figure 4. Thus, through the excitation frequency, one can know the resonant frequency and wavelength of the dominating modes of the instantaneous displacement field.

### 3.1. Simulation of Instantaneous Wave Field on the Damaged Plate

**Figure 5:**
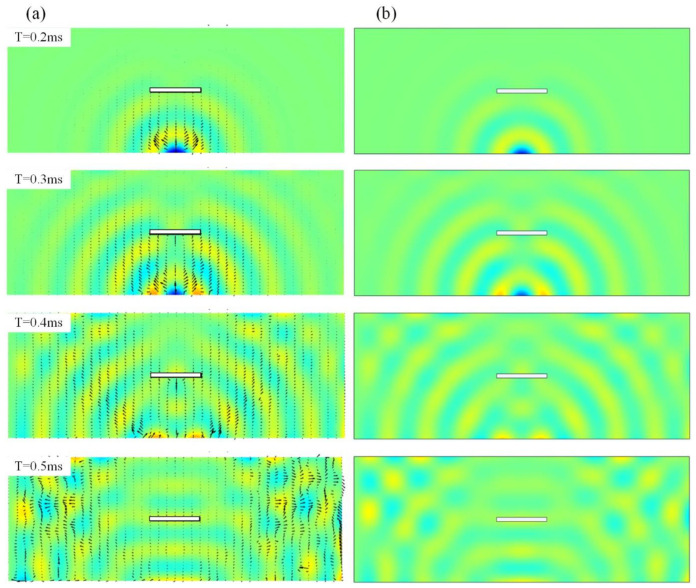
The full-field displacement response within 0.2 to 0.5 ms of the damaged plate (**a**) the semi-analytical method with the power transmission paths. (**b**) The FEM methods.

**Figure 6:**
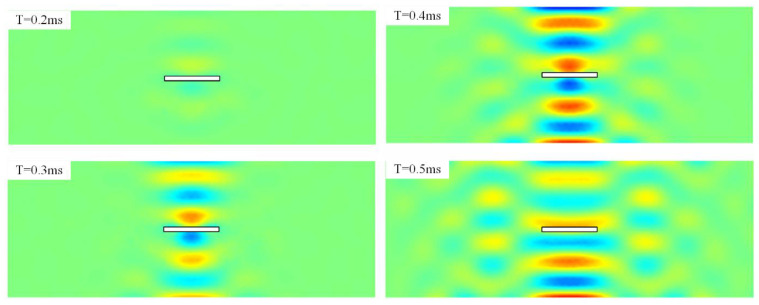
The subtracted full-field displacement response within 0.2 to 0.5 ms by the baseline subtraction method.

Figure 5 shows the simulated instantaneous wave field on the damaged plate. Good correspondence can be seen between the proposed semi-analytical method and the FEM. We can see the energy transport vectors are reduced when the instantaneous waves pass behind the slot. To separate the damaged information for clear observation, we then substrate the wave fields on the intact plate from those on the damaged plate, and the result is shown in Figure 6. Diffraction of the scattered elastic waves from the slot can be clearly seen due to the fact that the wavelength of the excited wave is larger than the corresponding dimension of the slot, and thus, the baseline subtraction method amplifies the influence of the presence of the damage on the propagating waves [40]. The intensity of the incoming wave distributes to the reflection waves and the scattered waves. From Figure 6, we can see that the presence of the damage reduces the intensity of the incoming transient waves, and that of the reflection wave is increased after performing the baseline subtraction method, and an antisymmetric displacement pattern can be observed. The pattern of the scattered waves follows the principle of diffraction, where a small obstacle would span the wave fields.

### 3.2. Simulation for the Pointwise Excitations

**Figure 7:**
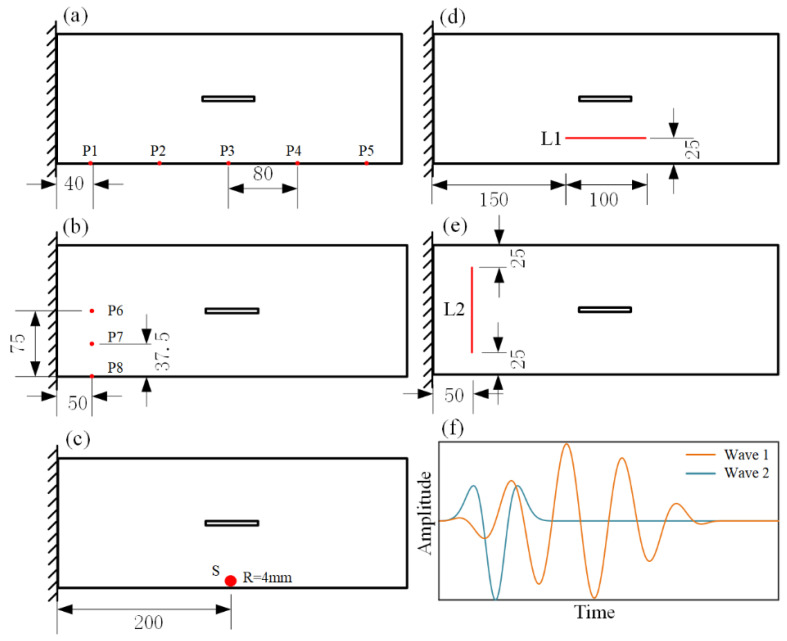
(**a**) Pointwise excitations at different horizontal locations. (**b**) Pointwise excitations at different vertical locations. (**c**) Surface excitation with a radius of R = 4 mm. (**d**) Horizonal line excitation. (**e**) Vertical line excitation. (**f**) Different temporal waveforms of excitations.

One advantage of the proposed semi-analytical method is that, when calculating the full-field instantaneous wave field on the damaged plate with different arrangements of the excitations, we only need to substitute different forms of excitations without the requirement of recalculating the vibration modes. Next, using the proposed semi-analytical method, we respectively model full-field transient Lamb waves on damaged plates with any spatial or temporal arrangement of excitations, as illustrated in Figure 7.

**Figure 8:**
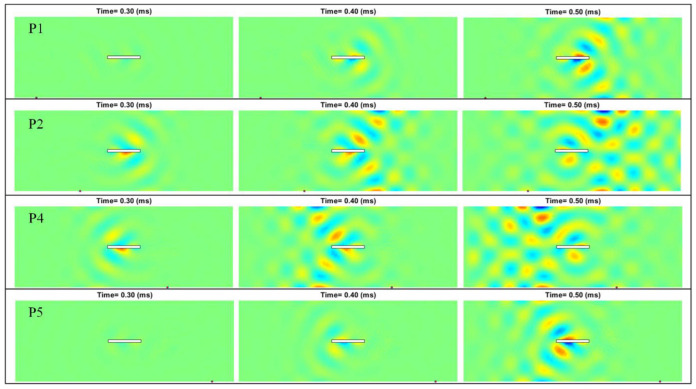
Differential instantaneous wave field when the pointwise excitations are at different locations.

First, we consider pointwise excitations at different locations, marked as P1~P5 in Figure 7a. Since different excitation locations lead to different arrival times and different incident angles of the Lamb waves, excitation locations will affect the instantaneous Lamb waves and the corresponding differential wave responses from the baseline subtraction method. Figure 8 shows the differential instantaneous wave fields when the excitations are at P1, P2, P4, and P5, where the results at P3 have been shown in Figure 6. When the Lamb waves are excited directly facing the long side of the slot (i.e., at P3), the diffraction of the Lamb waves is more significant. In addition, when exciting at P3, the differential instantaneous waves reflect the damage information much earlier than the excitations at other locations. For the instantaneous waves, the influence of the boundary condition is not significant in affecting the wave propagation, and we can see that the wave patterns are symmetric to the center axis in the *y* direction of the plate when the waves are excited at P1 and P5 or P2 and P4.

**Figure 9:**
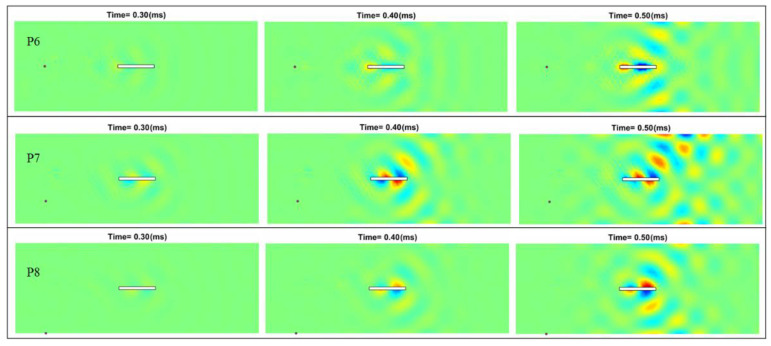
Differential instantaneous wave field when the pointwise excitations are at different locations from the left side of the damaged plate.

The simulation results when the pointwise excitations are at P6, P7, and P8 at the left side of the plate are shown in Figure 9. Since the damage “seen” by the incoming waves is smaller when the excitations are located normal to the short side of the slot, the intensity of the scattered waves is smaller compared to those in Figure 8. Note that unlike the results obtained when the excitation is at P6, the differential instantaneous wave field obtained when the excitation is at P8 is asymmetric to the slot due to the fact that the waves are asymmetrically reflected by the slot. These simulation results support the concept of identifying the damage information through the full-field instantaneous wave patterns by using pointwise excitations at different locations.

### 3.3. Simulation for the Surface or Linear Excitations

**Figure 10:**
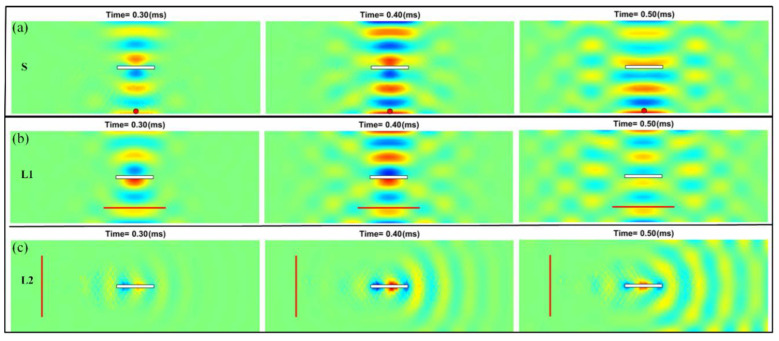
Differential instantaneous wave field when the excitations are (**a**) surface, (**b**) linear parallel to the long edge of the damaged plate, and (**c**) linear parallel to the short edge of the damaged plate.

Now, we consider more practical linear or surface excitations. We first replace the pointwise excitation located at P3 (as shown in Figure 7a and studied in Section 3.1) as a round surface excitation (as shown in Figure 7c) with a radius of 4 mm, a dimension identical to that of a commercial piezoelectric actuator. Under the surface excitation, the reflected A0 mode Lamb waves in front of the plate edges interfere with the incoming waves and distort the wave pattern from a circular one to that excited by the pointwise excitation [41]. Even generating a distorted circular wave pattern due to the presence of the boundary conditions, the differential instantaneous wave field shown in Figure 10a is similar to that when the pointwise excitations are at location P3 (i.e., Figure 6). Linear excitations are also practical, and the results of the linear excitations, as arranged in Figure 7d (parallel to the long edge of the plate) and Figure 6e (parallel to the short edge of the plate), are shown in Figure 10b,c. Similar to the pointwise excitations, the differential wave field in the damaged plate is also dependent on the relative locations between the excitation and the damage event as well as its geometry [4,12,38]. These simulations are efficiently carried out in a sense that the same group of temporal vibration modes is utilized.

### 3.4. Simulation for the Excitations with Specific Temporal Waveforms

After considering the simulation of different spatial excitations, we next consider excitations with different temporal waveforms. We consider the general five-cycle tone bursts, marked as Wave 1, at different frequencies and a Ricker wavelet, marked as Wave 2, as shown in Figure 7f. The expression of the Ricker wavelet is as follows:

(8) $$A_{2}(t)=(2{\left(\pi f_{0}(t-1/f_{0})\right)}^{2}-1)\exp(-{\left(\pi f_{0}(t-1/f_{0})\right)}^{2}).$$

The expression of the Ricker wavelet is substituted by Equation (4) to obtain the transient displacement solution of the flexural wave. However, unlike the wave source of the tone burst where a simpler closed form can be obtained, here, Simpson’s 1/3 rule is used to perform Duhamel’s integral.

**Figure 11:**
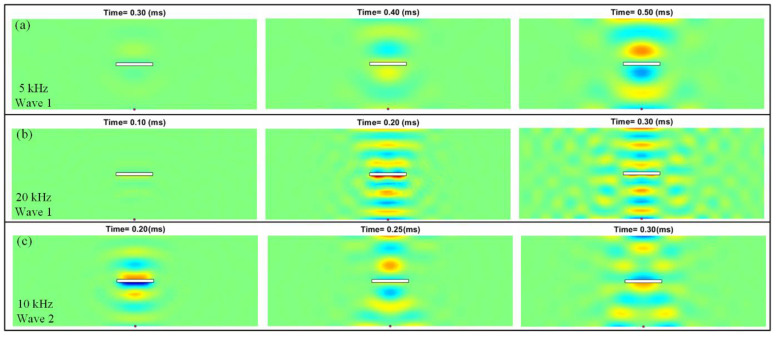
Differential instantaneous wave field when the excitations are (**a**) 5 kHz five-cycle tone burst, (**b**) 20 kHz five-cycle tone burst, and (**c**) the 10 kHz Ricker wavelet.

Figure 11a,b shows the differential wave field when the excitations are the 5 kHz and 20 kHz five-cycle tone bursts (i.e., Wave1), respectively. Obviously, dependent on the relation of the excitation frequency and the excited wavelength, the advantage of high-frequency excitations can be seen in damage detection. Without having to recalculate the vibration modes, we can efficiently simulate the full differential wave field, as shown in Figure 11c, by simply replacing the tone bursts as the Ricker wavelet (i.e., Wave2) with an operation frequency of 10 kHz.

## 4. Experimental Results and Discussions

To validate the proposed semi-analytical method for modeling the full-field transient Lamb waves, we performed experiments on an intact and a damaged aluminum plate (150×500×2 mm) utilizing the surface and the linear excitations. For the convenience of clamping one side of the plate, the long edge of the plate is longer than that of the plate considered in the previous modeling. For the surface excitation, the A0 mode Lamb waves were generated by a circular piezoelectric patch (Haiying Group, Wuxi, China), bonded on the location S (as shown in Figure 7c) of the plate. For the linear excitation, an array of the piezoelectric patches was bonded along the location L2 (as shown in Figure 7e). A 10 kHz five-cycle tone burst was excited by the piezoelectric patches using a function generator (DG4102, Rigol Technologies, Suzhou, China) and a voltage amplifier (Trek Model PZD350). A laser scanning vibrometer system (Polytec PSV-500) was used to measure the out-of-plane component of the propagating displacement wave fields of the intact plate and damaged plate over a grid of points.

**Figure 12:**
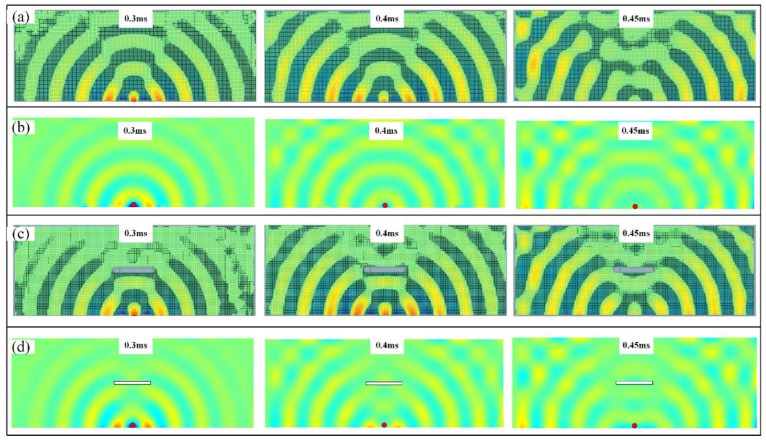
Instantaneous wave field under the surface excitations: (**a**) Experimental results of the intact plate; (**b**) semi-analytical results of the intact plate; (**c**) experimental results of the damaged plate; (**d**) semi-analytical results of the damaged plate.

**Figure 13:**
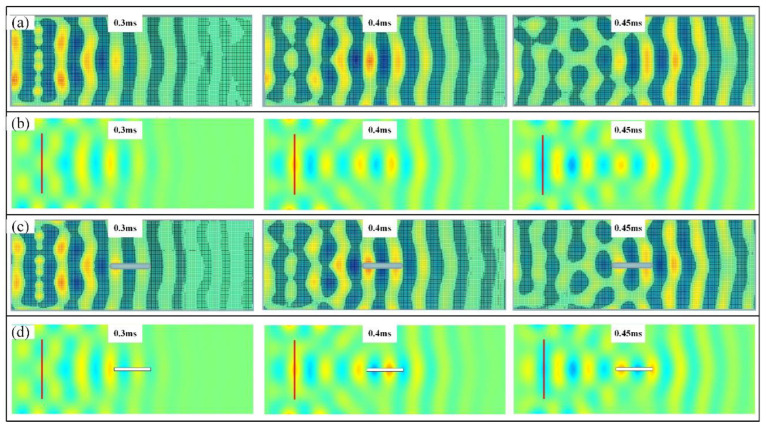
Instantaneous wave field under the linear excitations: (**a**) Experimental results of the intact plate; (**b**) semi-analytical results of the intact plate; (**c**) experimental results of the damaged plate; (**d**) semi-analytical results of the damaged plate.

The measured displacement fields at 10 kHz frequencies are given in Figure 12a,c and Figure 13a,c, which agree well with the results obtained from the semi-analytical method in Figure 12b,d and Figure 13a,c. From the wave patterns in Figure 12a,b, we can see the interference between the incoming and reflected waves due to the presence of the boundary when using a non-pointwise excitation source. In addition, from the experimental results in Figure 12, we can see that the slot disturbs the instantaneous incident wave fields. However, agreed with the analytical prediction, when the Lamb waves generated from the linear excitation meet the short edge of the slot, the incident wave is almost not disturbed due to the fact that the dimension of the damage is smaller than the wavelength.

## 5. Conclusions

In this paper, a semi-analytical method for modeling the propagation of Lamb waves based on the vibration characteristics of the intact or damaged plate is proposed. Using the same group of the temporal vibrational modes, the proposed method can model the full-field transient Lamb waves efficiently with any forms of excitations, where the temporal resolution relies on the number of the vibration modes. As an additional advantage, the wave fields expressed as the superposition of the vibration modes can be transformed into other physical quantities without having to perform any additional numerical post-processing operations. As an application, a power flow analysis is employed in this work to reveal the transmission paths of the propagating waves on the damaged plate. In addition to demonstrating the modeling capability of the semi-analytical method, we also examine its role in structure health monitoring on a classic damage model by combing the baseline subtraction method. After comparing the analytical results with the finite element method simulation, the proposed semi-analytical method is validated by a non-contact scanning Laser Doppler Vibrometer (LDV) system.

## Figures and Tables

**Table 1:** Comparison of the computational time between the FEM and semi-analysis method.

Sampling Time Δ*t*	FEM (Time-Dependent)	Semi-Analytical Method
3 μs	159 s	241.4 s (235 + 6.4)
2 μs	242 s	242.4 s (235 + 7.4)
1 μs	462 s	244.1 s (235 + 9.1)
0.5 μs	855 s	247.0 s (235 + 12.0)

## Data Availability

The data that support the findings of this study are available from the corresponding author upon reasonable request.
